# Stacking ensemble learning model to predict 6-month mortality in ischemic stroke patients

**DOI:** 10.1038/s41598-022-22323-9

**Published:** 2022-10-17

**Authors:** Lee Hwangbo, Yoon Jung Kang, Hoon Kwon, Jae Il Lee, Han-Jin Cho, Jun-Kyeung Ko, Sang Min Sung, Tae Hong Lee

**Affiliations:** 1 Department of Radiology, Pusan National University Hospital, Gudeokro 179, Seogu, Pusan, 49241 South Korea; 2 Department of Neurology, Pusan National University Hospital, Pusan, 49241 South Korea; 3 Department of Neurosurgery, Pusan National University Hospital, Pusan, 49241 South Korea; 4 Biomedical Research Institute, Pusan National University Hospital, Pusan, 49241 South Korea; 5 College of Medicine, Pusan National University, Yangsan, 50612 South Korea

**Keywords:** Data processing, Programming language

## Abstract

Patients with acute ischemic stroke can benefit from reperfusion therapy. Nevertheless, there are gray areas where initiation of reperfusion therapy is neither supported nor contraindicated by the current practice guidelines. In these situations, a prediction model for mortality can be beneficial in decision-making. This study aimed to develop a mortality prediction model for acute ischemic stroke patients not receiving reperfusion therapies using a stacking ensemble learning model. The model used an artificial neural network as an ensemble classifier. Seven base classifiers were K-nearest neighbors, support vector machine, extreme gradient boosting, random forest, naive Bayes, artificial neural network, and logistic regression algorithms. From the clinical data in the International Stroke Trial database, we selected a concise set of variables assessable at the presentation. The primary study outcome was all-cause mortality at 6 months. Our stacking ensemble model predicted 6-month mortality with acceptable performance in ischemic stroke patients not receiving reperfusion therapy. The area under the curve of receiver-operating characteristics, accuracy, sensitivity, and specificity of the stacking ensemble classifier on a put-aside validation set were 0.783 (95% confidence interval 0.758–0.808), 71.6% (69.3–74.2), 72.3% (69.2–76.4%), and 70.9% (68.9–74.3%), respectively.

## Introduction

Before the introduction of reperfusion therapies, such as intravenous thrombolysis or mechanical thrombectomy, about 14.5% to 20% of patients with acute ischemic stroke (AIS) succumbed to death within 1 month^1,2^. The indications for mechanical thrombectomy in AIS patients have continuously expanded in recent years^3–5^. Mechanical thrombectomy is, however, not without risk. The numbers needed to treat and harm are 8 and 92, respectively^6^. Starting mechanical thrombectomy is a difficult decision for a patient if she is not eligible by current indications^7^. Establishing an individualized mortality prediction model for AIS patients not undergoing reperfusion therapy at the time of presentation will be beneficial in aiding clinical decision-making.

There have been several mortality prediction models for AIS patients. While these models reported sufficient prediction accuracy, several of them predict the outcome based on variables that can only be assessed later in the disease course^8–10^. Even though the later-time clinical variables can promote its predicting capability, we hypothesized that having an accurate prediction model based exclusively on early clinical data at presentation can help decide hyperacute treatments.

Machine learning (ML) algorithms are now prevalent in medical research, including several mortality prediction models for ischemic stroke^9–12^. It enables researchers to develop accurate models. Using ML algorithms can also be beneficial where data exhibits significant collinearity. Stacking ensemble learning (SEL) is an algorithm structure consisting of more than one level of ML algorithms that constitutes the whole^13–15^. This type of ML is known to produce a more reliable model. To our knowledge, an SEL-based outcome prediction model for AIS is lacking.

The first International Stroke Trial (IST-1), published in 1997, compared the effects of aspirin and subcutaneous heparin and followed up for 6 months^16^. The anonymized dataset of this randomized controlled trial is made public^17^. The IST-1 was a large-scale trial and had a concise set of variables for randomization. During the study period in the 1990s, neither thrombectomy nor intravenous thrombolysis was widespread. Therefore, we saw IST-1 as an excellent dataset for evaluating 6-month mortality in AIS patients who have foregone reperfusion therapies.

This study aims to develop a robust model for 6-month mortality prediction in AIS patients who did not undergo reperfusion therapy using only a concise set of hyperacute-phase clinical data of the IST-1 with the help of stacking ensemble ML.

## Methods

### Ethical statement

This data-driven study followed regional regulations and ethical guidelines issued by the South Korean government^18^.

### Study design and dataset pre-processing

We used a publicly available anonymized dataset from the IST-1^16,17^.

The feature selection process was done by agreement of the stroke neurologists and neuro-interventionalists among the authors, who chose easily assessable variables at the initial workup. The list of all included variables is as follows: age, sex, level of consciousness at presentation, presence of wake-up stroke, underlying atrial fibrillation, visible infarction on computed tomography, heparinization within 24 h, aspirin administration within 3 days, systolic blood pressure, presence of deficits (including face, upper and lower extremities, dysphasia, hemianopsia, visuospatial disorder, and other neurological deficits), and aspirin or heparin administration at presentation. Age and systolic blood pressure were continuous variables; sex and level of consciousness were categorical; all other variables were binomial. We selected these 18 variables before any analysis, and its purpose was to capture clinically meaningful information while minimizing possible overfitting during the training of ML algorithms. The selected variables or features for ML were deemed meaningful in mortality prediction in AIS patients.

The application of exclusion criteria established patients for ML analysis. The excluded patients include those who received subcutaneous unfractionated heparin 12,500 units twice daily, as this is not a routinely advocated treatment by current practice guidelines^7^. Since carotid endarterectomy or thrombolysis could potentially confound the outcome, those who underwent were ineligible. Patients with missing values in study variables or non-ischemic stroke were not included in the analyses to minimize uncertainty during ML training. The previous reports described the outcome of the original trial and its dataset^16,17^.

Then we divided the prepared data into training and validation sets in a seven-to-three ratio. The validation set was used for evaluating constructed ML classifiers at the final stage. This retained group served exclusively as an internal validation set and was strictly put aside during model development.

### Base algorithm development

Each base algorithm received input values of 18 variables and produced a mortality probability value used by an ensemble classifier. Seven base ML algorithms consist of K-nearest neighbor (KNN), extreme gradient boosting (XGB), support vector machine (SVM) with radial basis function kernel, Gaussian Naïve Bayes (NB), random forest (RF), artificial neural networks (ANN), and logistic regression (LR) classifiers. We aimed to make the final model robust while maximizing potential information gains using diverse classifiers. The rationale for base classifier selection was apparent prevalence in the medical literature and ease of implementation during model development.

Hyperparameters tend to impact the performance of ML models. Moreover, we searched for their optimal values exhaustively within a reasonable range. Theoretically, hyperparameter values have infinite combinations, and consequently, heuristic methods were employed. Several small batches of pilot tests revealed the rough boundaries for hyperparameter values. Within practical limits, the range of any given hyperparameter was as wide as possible, while the testing points were as dense as feasible.

**Figure 1:**
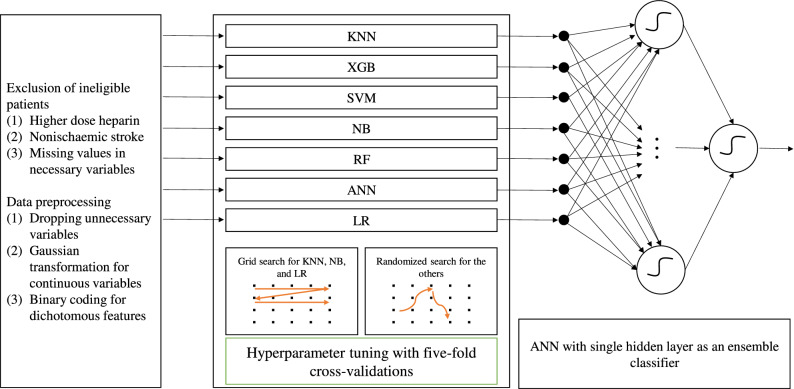
Design of stacking ensemble learner. Seven base classifiers are hyperparameter-tuned individually, and each produces a prediction value. The seven outcome prediction values are input variables for the stacking ensemble learner. KNN: k-nearest neighbours; XGB: extreme gradient boosting; SVM: support vector machine; NB: Naïve Bayes; RF: random forests; ANN: artificial neural networks; LR: logistic regression.

Grid or randomized search of hyperparameters with five-fold cross-validation yielded seven classifiers (Fig. 1). The former uses an exhaustive evaluation of all possible combinations of hyperparameters within a given hyperspace; the latter takes advantage of a randomized search of the hyperspace to minimize training time while preserving accuracy. Five-fold cross-validation enabled the training of base ML methods with the put-aside validation dataset unused during the process. Grid search identified the best hyperparameters for KNN, NB, and LR. Randomized search fine-tuned hyperparameters for the rest. The numbers of iterations for the randomized search of hyperparameters in XGB, SVM, RF, and ANN classifiers were 1024, 4096, 2048, and 4096, respectively. Small-batch pilot tests produced an approximate time required per iteration of an algorithm, and we used this as a guide to determine the numbers.

The hyperparameter space for the KNN model was k value for a k-nearest neighbor of integers 1 through 500; for XGB consists of learning rate between 10^–2^ to 10^–0.5^, maximum depth between 2 and 10, minimum child weight of 1 through 300, subsampling rate between 0.2 and 1.0, column sampling rate by the tree from 0.2 to 1.0, and the number of estimators between 50 and 1000; for SVM with radial basis function kernel were C value from 10^–2^ to 10^3^, and gamma between 10^–4^ and 10^4^; for NB was variable smoothing between 10^–15^ and 10^1^; for RF, the presence of bootstrap, maximum depth from 1 to 20, the maximum features be either automatic or square-root of the input features, minimum samples leaf between 1 and 10, and the minimum samples split between 1 and 10; for ANN was the number of nodes in a single hidden layer between 2 and 20, all available activation functions, solver functions, alpha parameter, and learning rate either be constant or adaptive; for LR, all available solver functions, and C value of 10^–8^ to 10^3^.

### Stacking ensemble algorithm development

We approached baseline ML algorithms agnostically. It was difficult to predict the best performer before validation, which was the main reason for choosing an SEL-based model over others. We constructed an ANN with a single hidden layer to accept probability values generated by seven individual base algorithms. Since the seven base classifiers would likely have collinearity, we assumed an ANN would be more suitable over regression models. Five nodes in a single hidden layer constituted a stacking ensemble classifier. The choice of the number of nodes was arbitrary. An underlying assumption was that this single-layer ANN would work as a universal approximating function^19,20^. Another conjecture was that limiting the number would prevent overfitting. A stratified fivefold cross-validation model evaluated the candidate models. This approach was topologically similar to a deep neural network when combined with the base ANN classifier.

### Algorithm implementation

We developed both base and ensemble ML models using the Scikit-learn (version 1.0.1) and XGBoost (version 1.5.0) library on Python (version 3.9.7)^21,22^.

### Evaluation of classifiers

We compared the train and validation sets using Pearson’s χ2 test or Student’s t-test for dichotomous and continuous variables. The level of significance, α, was set to 0.05.

The train and test sets were evaluated upon completing the ensemble learning. The evaluation metrics for each hyperparameter-tuned classifier and the final ensemble algorithm included an area under the receiver-operating characteristics (AUROC), accuracy, sensitivity, specificity, positive predictive value (PPV), negative predictive value (NPV), positive likelihood ratio (LR+), and negative likelihood ratio (LR−), F_1_-score with 95% confidence interval (CI). A receiver-operating characteristics (ROC) analysis using the final probability value and the actual mortality revealed the threshold value for probability outcome maximizing Youden's J statistics (sensitivity + specificity − 1) divided by the absolute difference of the sensitivity and specificity plus 0.01 to prevent division by zero. We added the divisor term to control the excessive gain of sensitivity or specificity at the expense of the other, which was frequently observed during pilot tests using a small number of iterations. Bootstrapping 10,000 times produced 95% CI for the performance metrics and enabled violin plot of the measured performance metrics. The AUROC, accuracy, and population-independent metrics, including sensitivity, specificity, LR+, and LR− were plotted for seven base classifiers and the final ensemble learner using a Python library Matplotlib^23^.

## Results

### Baseline analysis of study patients in test and validation sets

**Figure 2:**
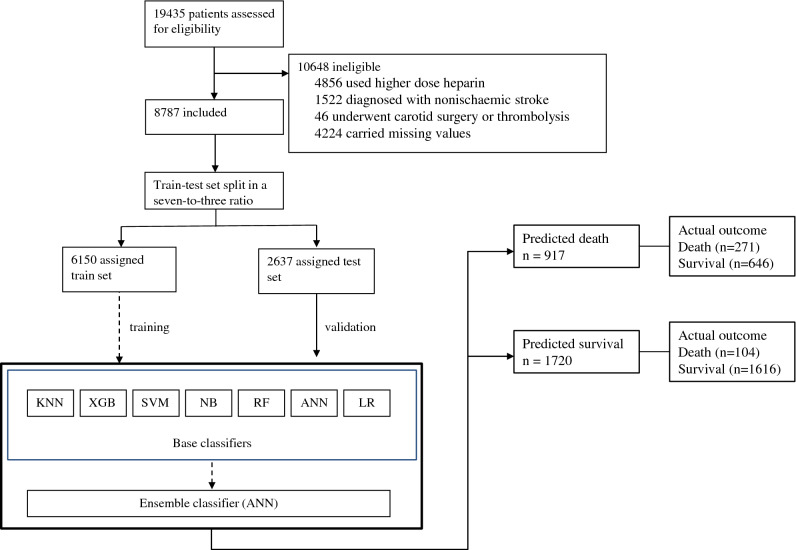
Flow of study patients. The confusion matrix of the final stacking ensemble learning produced the predicted number. The put-aside validation set evaluated the models after complete training.

Among 19,435 patients in the IST-1 dataset, excluded from the ML training were 10,648 for the following reason: 4856 for higher dose heparin, 1522 for non-ischemic stroke as a final diagnosis, 46 for carotid surgery (endarterectomy) or thrombolysis, 4224 for missing values in study variables. A seven-to-three ratio split of 8787 study subjects between train and test sets resulted in 6150 assigned in the former and 2637 in the latter (Fig. 2). Comparing the study variables of the groups revealed a difference in proportion regarding dysphasia (p = 0.0399), while the other variables showed similar characteristics (Table 1).

**Table 1:** Summary of clinical variables of train and validation sets.

	Train set	Validation set	P value
Age (SD), years	70.4 (11.5)	70.1 (11.9)	0.2896
Sex (female)	2618 (42.6%)	1177 (44.6%)	0.07716
Altered consciousness (drowsy or sunconscious)	706 (11.1%)	294 (11.4%)	0.6813
Wake-up stroke	1888 (30.7%)	818 (31.0%)	0.7845
Atrial fibrillation	894 (14.5%)	343 (13.0%)	0.0635
Visible infarction on computed tomography	1981 (32.2%)	874 (33.3%)	0.4063
Heparin within 24 h of visit	150 (2.4%)	67 (2.5%)	0.8363
Aspirin within 3 days of visit	1304 (21.2%)	523 (19.8%)	0.1551
Systolic blood pressure (SD), mmHg	160.4 (27.4)	160.7 (28.2)	0.6780
**Deficits**			
Facial	4318 (70.2%)	1848 (70.1%)	0.9217
Upper extremity	5188 (84.4%)	2243 (85.1%)	0.4228
Lower extremity	4478 (72.8%)	1956 (74.1%)	0.1952
Dysphasia	2225 (84.4%)	893 (33.9%)	0.0399
Hemianopsia	1034 (16.8%)	431 (16.3%)	0.6108
Visuospatial disorder	945 (15.4%)	395 (15.0%)	0.6674
Brainstem-cerebellar	746 (12.1%)	333 (12.6%)	0.5377
Other	376 (6.1%)	137 (5.2%)	0.1024
Heparin administered	2058 (33.5%)	887 (33.6%)	0.8941
Aspirin administered	3098 (50.4%)	1294 (49.1%)	0.2729
Death at 6 months	910 (14.8%)	375 (14.2%)	0.5045

Tabulated data are number of patients for binary variables and mean for continuous variables. The p values are calculated with either Pearson’s χ^2^ test or Student’s t-test.

### Developed algorithms

Seven base classifiers and the final ensemble model are freely available on an online repository (see data availability statement).

### Predictive performance of individual and ensemble models

**Figure 3:**
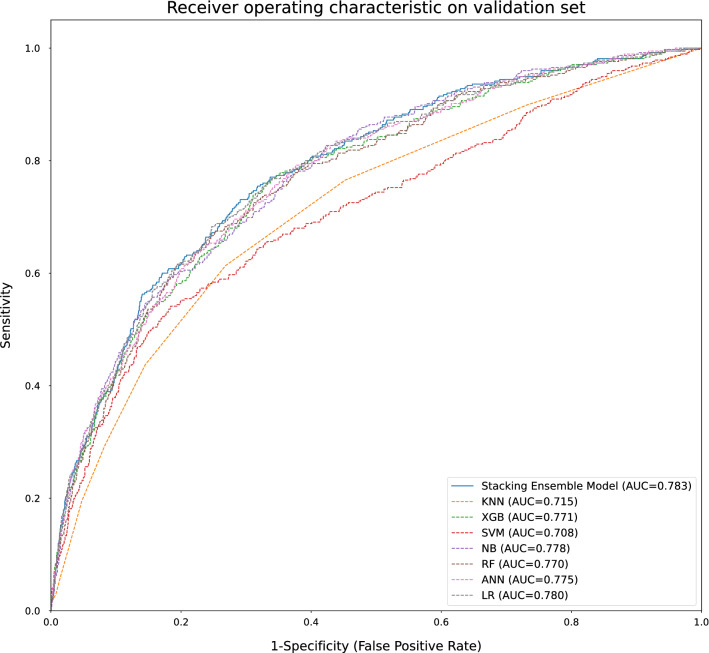
Receiver operating characteristics curve for seven base and ensemble learners. KNN: k-nearest neighbors; XGB: extreme gradient boost; SVM: support vector machine; NB: Naïve Bayes; RF: random forests; ANN: artificial neural networks, LR = logistic regression.

The ROC of seven individual ML algorithms and the ensemble learner on the validation set were plotted for analysis. It showed similar performance of the XGB, NB, RF, LR, and ANN to the SEL with their respective AUROC of 0.771 (95% confidence interval [CI] 0.744–0.796), 0.778 (0.753–0.803), 0.770 (0.743–0.795), 0.780 (0.752–0.808), and 0.775 (0.749–0.800), while the k-nearest neighbors (KNN) and support vector machine (SVM) models performed poorly with 0.715 (0.686–0.743) and 0.708 (0.678–0.739), respectively (Fig. 3).

The final stacking ensemble model used all seven base ML algorithms. It resulted in AUROC, accuracy, sensitivity, specificity, PPV, and NPV of 0.783 (95% CI 0.758–0.808), 71.6% (69.3–74.2), 72.3% (69.2–76.4), 70.9% (68.9–74.3), 29.6 (26.6–33.1), and 94.0 (93.0–95.0), respectively, when tested on the validation set (Table 2). The LR+ and LR− were 2.48 (2.29–2.87), and 0.391 (0.330–0.437).

**Table 2:** Model performance on train and validation set of stacking ensemble machine learning.

	Train set	Validation set
AUROC	0.797 (0.782–0.813)	0.783 (0.758–0.808)
Accuracy	0.728 (0.707–0.742)	0.716 (0.693–0.742)
Sensitivity	0.719 (0.703–0.745)	0.723 (0.692–0.764)
Specificity	0.732 (0.705–0.744)	0.709 (0.689–0.743)
Positive predictive value	0.316 (0.291–0.338)	0.296 (0.266–0.331)
Negative predictive value	0.937 (0.931–0.944)	0.940 (0.930–0.950)
Positive likelihood ratio	2.69 (2.42–2.86)	2.48 (2.29–2.87)
Negative likelihood ratio	0.384 (0.348–0.414)	0.391 (0.330–0.437)
F_1_ score	0.439 (0.413–0.463)	0.420 (0.387–0.457)

Proportion or ratio (bootstrapped 95% CI).

### Comparison of individual and ensemble models on bootstrapped metrics

**Figure 4:**
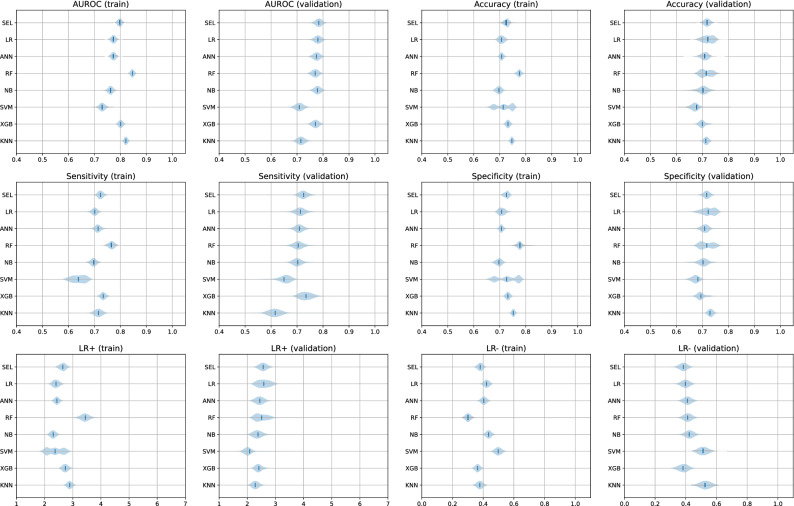
Violin plots of bootstrapped metrics of AUROC, accuracy, sensitivity, specificity, LR+, and LR−. AUROC: area under the receiver operating characteristics curve; LR+: positive likelihood ratio; LR−: negative likelihood ratio.

The diagnostic performance of individual base learners and ensemble classifiers on train and validation sets showed a slight decrease in performance in most models on violin plots of bootstrapped metrics (Fig. 4). The most pronounced decline was for the RF model, with its AUROC decreasing from 0.846 (95% CI 0.832–0.860) to 0.770 (0.744–0.795). The SEL, LR, ANN, and XGB models fared well on the validation set, with the AUROC values changing from 0.797 (0.782–0.813), 0.773 0.757–0.790), 0.774 (0.757–0.790), and 0.801 (0.785–0.817) on the training set to 0.783 (0.758–0.808), 0.780 (0.754–0.805), 0.775 (0.749–0.800), 0.771 (0.744–0.796) on the validation, respectively. It was interesting to observe the instability of SVM on the train set when bootstrapped, especially in terms of accuracy and specificity, where it shows three peaks of bootstrapped metrics with a wider range of bootstrapped distribution, which was not obvious on the validation set. On the other hand, the LR and RF models revealed a similar widening of bootstrapped accuracy and specificity on the validation set with two peaks. The final SEL model did not show such deviations in metrics. All its metrics on both sets showed a single peak with a stable distribution width.

## Discussion

This study demonstrated a final ensemble model with an AUROC of 0.783 for a 6-month mortality prediction. From a clinical perspective, making this prediction requires only a part of the National Institute of Health Stroke Scale (NIHSS) assessment, an electrocardiogram, and a brief review of current medications. This concise set of clinical variables alone resulted in sensitivity and specificity of 71.6% and 72.3%. These results are somewhat insufficient to decide on reperfusion therapy in AIS patients. However, this model can add information to clinical decisions considering LR+ and LR− of 2.48 and 0.391.

When applied to a research setting, it would serve as a classification scheme for AIS patients, especially when its primary outcome involves 6-month mortality. We succinctly chose clinical variables, which can benefit retrospective studies as these variables are universally assessed in the current practice environment.

Several reports of outcome prediction models for acute ischemic strokes and a few ML-derived models have been published recently^8–10,24^. Moreover, our model is unique in that the required clinical variables are straightforward, and all variables are assessable at the time of presentation. We summarized the hyperacute assessability of selected mortality prediction models for AIS patients in Table 3.

**Table 3:** Comparison of selected mortality prediction models for AIS patients.

	Algorithms used	Validations	Hyperacute applicability	Predicting outcomes	Reported AUROC
Current study	SEL	Internal validation	Yes	6-month mortality	0.783
Saposnik et al.	Integer scoring system	Internal and incomplete external validations (half of the external set used for calibration)	Yes	30-day and 1-year mortality	0.790 (30-day)
					0.782 (1-year)
Eaton et al.	NB	Internal validation	No	7- and 93-day mortality	0.858 (7-day)
					0.807 (93-day)
Fernandez-Lozano et al.	RF	Internal validation	No	3-month morbidity and mortality	0.703 (3-month morbidity)
					0.899 (3-month mortality)
Abedi et al.	LR, RF, XGB	Internal validation	No	1-, 3-, 6-, 12-, 18-, 24-month mortality	0.82 (1-month; RF)
					0.80 (6-month; RF)
					0.77 (12-month; XGB)
Lin et al.	RF, SVM, ANN, hybrid ANN	Internal validation	No	90-day morbidity	0.972 (RF)
					0.971 (SVM)
					0.969 (ANN)
					0.974 (hybrid ANN)

Hyperacute applicability means all selected features are assessable at the time of initial presentation.

Saposnik et al. proposed a useful integer scoring system called ‘IScore’ based on multivariate analysis^24^. Their work reported AUROC of 0.852 and 0.840 for 30-day and one-year mortality prediction on an internal validation set and 0.790 and 0.782 when externally validated. The input variables for their model were past medical history, comorbid conditions, preadmission disability, and serum glucose level. A head-to-head comparison with our study is not feasible as the SEL model was tested with internal validation only. However, the SEL model of this study is different in the timeframe it aimed to predict, and the variables used. In our SEL model, the input variables were those only available within minutes of presentation. This simple set of variables does not include any laboratory study. Consequently, it is beneficial when a prompt treatment decision is required. We expect our model would complement their scoring system.

Another interesting study by Easton et al. used UK Glucose Insulin in Stroke Trial database and reported an internally validated AUROC of 0.807 for 93-day mortality prediction using a Naïve Bayes classifier^10^. The study variables included laboratory tests such as plasma sodium/potassium concentration, serum urea, and creatinine. These laboratory tests typically take more than an hour to complete, and a current clinical guideline state that only blood glucose measurement can precede intravenous thrombolysis. The diagnostic performance of our model is comparable to this without employing any laboratory test.

An RF-based prediction model by Fernandez-Lozano et al. reported an excellent AUROC of 0.909 for 3-month mortality prediction for AIS patients^9^. Their model incorporated laboratory studies, along with 24-h and 48-h NIHSS. This RF-based model could be an important tool to assess AIS patients after 48 h, but this model is not feasible in a hyperacute setting.

One study by Abedi et al. examined and compared three ML algorithms, LR, RF, and XGB, and predicted a 6-month AUROC of 0.80 with the RF algorithm when internally validated^25^. This ML study also incorporated various laboratory tests as input variables and is likewise less applicable in a hyperacute clinical scenario.

A study with four ML models predicting AIS mortality at 90 days by Lin et al. reported AUROC for RF, SVM, ANN, and custom-designed hybrid artificial neural networks of 0.972, 0.971, 0.969, and 0.974, respectively^8^. They used clinical data for up to 30 days since ictus among 35,798 AIS patients from the Taiwanese Stroke Registry. These values are probably the best so far regarding AIS mortality prediction. This study is an excellent example that ML algorithms can extract information from data. It also incorporated subacute-phase variables and is not built for prediction based on clinical variables at presentation.

Stacking ensemble algorithm is a generic term applied to any machine learning method using more than one layer of classifiers^14,15^. Stacking can function as an error-correcting and bias-reducing scheme. ANN is known to approximate any continuous function. We found several successful examples of stacking ensemble algorithms implemented for medical data, such as intensive care unit mortality prediction and classification of Parkinson’s disease^26,27^. However, our proposed model is the first SEL developed for stroke outcome prediction to our best knowledge.

Choosing ANN as an ensemble algorithm can harness its approximating power to fit arbitrary relationships between probability values generated by each base classifier. Besides, the seven predicting variables are likely to exhibit collinearity. Therefore, we opted for ANN over logistic regression as an ensemble classifier and regarded it as a universal approximating function^19,20^. Topologically, this resembles deep neural networks as another ANN is among the base classifiers.

We chose seven algorithms as individual base classifiers. It is a potential benefit of ensemble learning to minimize the case of overfitting during training. This advantage may be cautiously attributable to differences in algorithmic mechanisms of each base learner. KNN algorithm predicts mortality based on the distance calculated in the hyperspace of clinical data^28^. For a patient with an unknown outcome, value k is predefined, and k-closest patients' data with known survival status are used to predict her mortality. XGB is a decision tree algorithm with gradient boosting^21^. The decision trees are sequentially generated for clinical data by minimizing the error of mortality prediction of the previous decision tree. During this process, the gradient descent algorithm makes this revision effective. XGB further improves this process by adopting several additional techniques. SVM tries to delineate a boundary between two groups of patients, either dead or alive, at 6 months^28^. This boundary is set to maximize the distance to the nearest patient data. NB algorithm uses the Bayes' theorem as if each variable is linearly independent^28^. Some variables may be linearly dependent. Nevertheless, this makes prediction modeling less complicated and computationally advantageous. RF algorithm is another decision tree algorithm, where each tree independently predicts the outcome using a portion of variables^28^. These trees form a collective decision-making scheme. ANN is a biomimetic machine learning algorithm that resembles a biological neuronal network^29^. Perceptron, a neuron analog, is gathered and layered to produce predicting algorithm. An LR classifier was also in the base learner. We intended to maximize information extraction from the dataset while avoiding overfitting or instability of algorithms by this ensemble learning.

The violin plots of bootstrapped performance metrics depicted this theoretical advantage. The final SEL model only marginally underperformed on the validation set, even though it has a clinical variable with a statistically significant difference. Moreover, all the bootstrapped metrics of the SEL revealed a single peak around the reported value. We believe this is indirect evidence of the stability of our model.

This study is not without limitations.

First, only a put-aside internal validation set tested the model. We chose an SEL approach to make the model as robust as possible, keeping in mind this limitation.

Second, our criteria excluded more than half of the patients from the IST-1 dataset. We saw this exclusion as technically necessary to incorporate all seven machine learning models as base learners. At the same time, it could have decreased the overall information extracted from the dataset. Even after excluding patient data with missing values, all seven base algorithms learned effectively.

Third, newer medications are now widely used, including antidyslipidemic agents, oral hypoglycemic medications, newer antiplatelet drugs, and direct oral anticoagulants. The differences in medication status may impact the overall model performance. Therefore, a direct extrapolation of this model to current AIS patients requires caution. However, as large-scale studies afterward included reperfusion treatments, the IST-1 dataset remained the only source for our purpose, i.e., predicting mortality when a patient skips systemic or endovascular reperfusion. In the same regard, it is unlikely to see a future prospective study not incorporating reperfusion therapies.

Fourth, the proposed SEL model used in this study itself is not technically new. However, the minimized features selected for the training to predict AIS outcomes were rare before our work. Subsequently, our model has the strength of clinical applicability at the time of initial presentation.

Despite these limitations, our model resulted in comparable diagnostic performance to the previous reports with a more concise set of variables that is obtainable with ease at presentation. To our knowledge, this work is the first to report SEL to predict AIS outcomes. We hope this model contributes to a decision process in practice and clinical research.

## Data Availability

The dataset we have used is from an openly available source. The implementation of our algorithms is freely available for research on a Github repository (https://github.com/lhwangbo/m-ist).

## References

[1] Sacco RL, Wolf PA, Kannel WB, McNamara PM (1982). Survival and recurrence following stroke. The Framingham study. Stroke.

[2] Brønnum-Hansen H, Davidsen M, Thorvaldsen P (2001). Long-term survival and causes of death after stroke. Stroke.

[3] Bracard S (2016). Mechanical thrombectomy after intravenous alteplase versus alteplase alone after stroke (THRACE): A randomised controlled trial. Lancet Neurol..

[4] Nogueira RG (2018). Thrombectomy 6 to 24 hours after stroke with a mismatch between deficit and infarct. N. Engl. J. Med..

[5] Albers GW (2018). Thrombectomy for stroke at 6 to 16 hours with selection by perfusion imaging. N. Engl. J. Med..

[6] Church EW, Gundersen A, Glantz MJ, Simon SD (2017). Number needed to treat for stroke thrombectomy based on a systematic review and meta-analysis. Clin. Neurol. Neurosurg..

[7] Powers WJ (2019). Guidelines for the early management of patients with acute ischemic stroke: 2019 update to the 2018 guidelines for the early management of acute ischemic stroke a guideline for healthcare professionals from the American Heart Association/American Stroke A. Stroke.

[8] Lin CH (2020). Evaluation of machine learning methods to stroke outcome prediction using a nationwide disease registry. Comput. Methods Programs Biomed..

[9] Fernandez-Lozano C (2021). Random forest-based prediction of stroke outcome. Sci. Rep..

[10] Easton JF, Stephens CR, Angelova M (2014). Risk factors and prediction of very short term versus short/intermediate term post-stroke mortality: A data mining approach. Comput. Biol. Med..

[11] Eun MY, Jeon ET, Seo KD, Lee D, Jung JM (2021). Reperfusion therapy in acute ischemic stroke with active cancer: A meta-analysis aided by machine learning. J. Stroke Cerebrovasc. Dis..

[12] Ntaios G (2021). Machine-learning-derived model for the stratification of cardiovascular risk in patients with ischemic stroke. J. Stroke Cerebrovasc. Dis..

[13] Jin, L. P. & Dong, J. Ensemble deep learning for biomedical time series classification. *Comput. Intell. Neurosci.* **2016**, 6212684 (2016). 10.1155/2016/6212684 PMC5048093 27725828

[14] Wolpert DH (1992). Stacked generalization. Neural Netw..

[15] Divina F, Gilson A, Goméz-Vela F, Torres MG, Torres JF (2018). Stacking ensemble learning for short-term electricity consumption forecasting. Energies.

[16] Sandercock PAG (1997). The International Stroke Trial (IST): A randomised trial of aspirin, subcutaneous heparin, both, or neither among 19 435 patients with acute ischaemic stroke. Lancet.

[17] Sandercock PAG, Niewada M, Członkowska A (2011). The International Stroke Trial database. Trials.

[18] Interdepartmental Commitee. *Ethical Guidelines on Artificial Intelligence for Humans*. (2020).

[19] Cybenko G (1989). Approximation by superpositions of a sigmoidal function. Math. Control Signals Syst..

[20] Hornik K (1991). Approximation capabilities of multilayer feedforward networks. Neural Netw..

[21] Chen, T. & Guestrin, C. XGBoost: A scalable tree boosting system. In *Proceedings of the ACM SIGKDD International Conference on Knowledge Discovery and Data Mining* Vol. 13–17-Augu, 785–794 (Association for Computing Machinery, 2016).

[22] Pedregosa F (2011). Scikit-learn: Machine learning in python. J. Mach. Learn. Res..

[23] Hunter JD (2007). Matplotlib: A 2D graphics environment. Comput. Sci. Eng..

[24] Saposnik G (2011). IScore: A risk score to predict death early after hospitalization for an acute ischemic stroke. Circulation.

[25] Abedi V (2021). Predicting short and long-term mortality after acute ischemic stroke using EHR. J. Neurol. Sci..

[26] El-Rashidy N, El-Sappagh S, Abuhmed T, Abdelrazek S, El-Bakry HM (2020). Intensive care unit mortality prediction: An improved patient-specific stacking ensemble model. IEEE Access.

[27] Yang Y (2021). Classification of Parkinson’s disease based on multi-modal features and stacking ensemble learning. J. Neurosci. Methods.

[28] Maleki F (2020). Overview of machine learning part 1: Fundamentals and classic approaches. Neuroimaging Clin. N. Am..

[29] Le WT, Maleki F, Romero FP, Forghani R, Kadoury S (2020). Overview of machine learning: part 2: Deep learning for medical image analysis. Neuroimaging Clin. N. Am..

