# Author Correction: Stacking ensemble learning model to predict 6-month mortality in ischemic stroke patients

**DOI:** 10.1038/s41598-022-26732-8

**Published:** 2022-12-21

**Authors:** Lee Hwangbo, Yoon Jung Kang, Hoon Kwon, Jae Il Lee, Han‑Jin Cho, Jun‑Kyeung Ko, Sang Min Sung, Tae Hong Lee

**Affiliations:** 1 Department of Radiology, Pusan National University Hospital, Gudeokro 179, Seogu, Pusan, 49241 South Korea; 2 Department of Neurology, Pusan National University Hospital, Pusan, 49241 South Korea; 3 Department of Neurosurgery, Pusan National University Hospital, Pusan, 49241 South Korea; 4 Biomedical Research Institute, Pusan National University Hospital, Pusan, 49241 South Korea; 5 College of Medicine, Pusan National University, Yangsan, 50612 South Korea

Correction to: *Scientific Reports* 10.1038/s41598-022-22323-9, published online 17 October 2022

The original version of this Article contained an error in the Funding section.

“This work is funded by Pusan National University Research Grant 2020. The funder had no role in study design, data collection, software development, data analysis, data interpretation, writing of the report, or decision to submit the paper.”

now reads:

“This work was supported by a 2-Year Research Grant of Pusan National University. The funder had no role in study design, data collection, software development, data analysis, data interpretation, writing of the report, or decision to submit the paper.”

The original Article has been corrected.

